# Rational design of self-assembled RNA nanostructures for HIV-1 virus assembly blockade

**DOI:** 10.1093/nar/gkab1282

**Published:** 2021-12-30

**Authors:** Na Qu, Yachen Ying, Jinshan Qin, Antony K Chen

**Affiliations:** Department of Biomedical Engineering, College of Future Technology, Peking University, Beijing 100871, China; Department of Biomedical Engineering, College of Future Technology, Peking University, Beijing 100871, China; Department of Biomedical Engineering, College of Engineering, Peking University, Beijing 100871, China; National Biomedical Imaging Center, Peking University, Beijing 100871, China; Department of Biomedical Engineering, College of Future Technology, Peking University, Beijing 100871, China; Department of Biomedical Engineering, College of Engineering, Peking University, Beijing 100871, China; National Biomedical Imaging Center, Peking University, Beijing 100871, China; Department of Biomedical Engineering, College of Future Technology, Peking University, Beijing 100871, China; National Biomedical Imaging Center, Peking University, Beijing 100871, China

## Abstract

Many pathological processes are driven by RNA-protein interactions, making such interactions promising targets for molecular interventions. HIV-1 assembly is one such process, in which the viral genomic RNA interacts with the viral Gag protein and serves as a scaffold to drive Gag multimerization that ultimately leads to formation of a virus particle. Here, we develop self-assembled RNA nanostructures that can inhibit HIV-1 virus assembly, achieved through hybridization of multiple artificial small RNAs with a stem–loop structure (STL) that we identify as a prominent ligand of Gag that can inhibit virus particle production via STL-Gag interactions. The resulting STL-decorated nanostructures (double and triple stem–loop structures denoted as Dumbbell and Tribell, respectively) can elicit more pronounced viral blockade than their building blocks, with the inhibition arising as a result of nanostructures interfering with Gag multimerization. These findings could open up new avenues for RNA-based therapy.

## INTRODUCTION

Assembly of a human immunodeficiency virus type-1 (HIV-1) virus particle is a result of extensive interactions between the viral genomic RNA (gRNA) and several thousand molecules of the viral Gag protein, at the plasma membrane (PM). Apart from the highly specific interaction between a small number of Gag molecules and a relatively short stretch of gRNA termed Ψ that is responsible for selective gRNA packaging (1–4), the assembly process is thought to occur predominantly via nonspecific interactions between the rest of the Gag molecules and the longer remainder of gRNA, with gRNA serving as a scaffold to concentrate Gag and mediate its multimerization into a virus particle (5,6). Thus, in the absence of gRNA, Gag multimerization is not abolished, with a random assortment of long-stranded cellular RNAs serving as scaffolds to result in the generation of non-infectious, virus-like particles that are morphologically identical to an authentic virus particle (7,8). Consistent with the proposed role of nonspecific Gag-RNA interactions in HIV-1 assembly, we have recently shown that microRNAs when not mediating gene silencing can bind Gag in a sequence-independent manner, forming microRNA-Gag complexes that can disrupt gRNA or cellular RNA-mediated Gag multimerization and virus production (9–11).

The inhibition conferred by the naturally occurring small RNAs as shown in our previous studies has prompted the present study in which we aim to develop a tunable and modular anti-HIV-1 assembly platform based on RNA nanotechnology, which focuses on constructing functional nanodevices that self-assemble through base pairing and folding of multiple synthetic small RNA oligonucleotides (oligos) (12–15). Compared with naturally occurring RNAs, synthetic RNAs carry several advantages including controllable nucleotide compositions, shapes, sizes and chemical modifications. We began our investigation by first searching for a prominent synthetic small RNA ligand of Gag that can inhibit HIV-1 assembly without inducing gene silencing. This was followed by construction of multivalent ligand nanostructures that displayed enhanced anti-HIV assembly potency.

## MATERIALS AND METHODS

### Synthesis of oligos and self-assembled double and triple stem–loop nanostructures

All oligos used in this study (listed in Supplementary Table S1) were synthesized by Integrated DNA Technologies (Coralville, IA, USA). Synthesis of self-assembled double and triple stem–loop nanostructures, denoted as Dumbbell and Tribell, respectively, was performed following procedures similar to those described previously (16). In brief, the module oligos of Dumbbell and Tribell (Supplementary Table S1) were mixed in equimolar concentrations in 1× Tris buffer (100 mM NaCl, 50 mM Tris, pH 8.0), and heated to 95°C for 10 minutes (min) and slowly cooled to 25°C over 70 min using a PCR machine. Thereafter, the desired products were separated from free oligos or other potential byproducts on a 6% native PAGE gel run in 1× TBM buffer (89 mM Tris, 200 mM boric acid, and 2.5 mM MgCl_2_) at 90 V, with Low Range ssRNA Ladder (New England Biolabs) used as the molecular weight marker. The gel was then stained with SYBR^®^ Gold (Life Technologies) in 1× TBM buffer for 10 min and visualized using a ChemiDoc XRS+ imaging system (Bio-Rad). The desired gel band was recovered following procedures described previously (17), with modifications. In brief, each desired gel slice was cut from the gel, placed in a 1.5 ml Eppendorf tube and then crushed into small pieces using a Squisher-single (Zymo Research). Following addition of 500 μl of 1× Tris buffer, the mixture was placed on an Eppendorf thermomixer with agitation set at 300 rpm and temperature set at 25°C for 24 h. After centrifugation at 21 000 × g for 5 min at 25°C to remove the gel debris, the supernatant was recovered and then concentrated on an Amicon Ultra 0.5 ml centrifugal filter (3000 NMWL). A small aliquot was then taken to measure the concentration of the final products using a Nanodrop spectrophotometer (Thermo Fisher Scientific) and to check for purity using PAGE as described above. Only solutions containing self-assembled products with purity greater than 90% were used for further studies.

### Topographic imaging by atomic force microscopy

For topographic atomic force microscopy (AFM) imaging under liquid conditions, 15 μl of 30–50 nM RNA samples were deposited onto a freshly cleaved mica surface for 30 s, followed by addition of 8 μl of 100 mM MgCl_2_ and 40 μl of 20 mM NiCl_2_ onto the mica surface for 2 min. Images were collected with a BioScope Resolve^TM^ AFM system (Bruker, Billerica, MA, USA) in peak force tapping mode at room temperature. Silicon nitride probes (PeakForce-HiRs-F-B, Bruker) with a tip radius of 1.5 nm and a nominal spring constant of 0.12 N/m were used for imaging at a scanning rate of 1 Hz. All images were processed and analyzed using the NanoScope Analysis software (Bruker, version 1.8).

### Plasmids construction

Generation of the HIV-1 proviral constructs pNL43ΔPolΔEnv-Gag, pNL43ΔPolΔEnv-Gag-EGFP, pNL43ΔPolΔEnv-Gag-mEOS2, pNL43ΔPolΔEnv-ΔNC-Gag, pNL43ΔPolΔEnv-ΔNC-Gag-EGFP and pNL43ΔPolΔEnv-Gag_ZiL_ has been described previously (9). To generate pNL43ΔPolΔEnv-Gag_ZiL_-EGFP, pNL43ΔPolΔEnv-Gag_ZiL_ was digested using SpeI and EcoRI restriction enzymes, and the resulting fragment was inserted into pNL43ΔPolΔEnv-Gag-EGFP digested using the same restriction enzymes. See Supplementary Figure S1 for schematic representation of each construct.

### Cell culture and plasmid transfection

HeLa cells were cultured in Dulbecco's modified Eagle's medium (DMEM, LONZA), supplemented with 10% (vol/vol) FBS (PAN™ Biotech), 1× GlutaMAX™ (Thermo Fisher) at 37°C, 5% (vol/vol) CO_2_, and 90% relative humidity. Jurkat cells were cultured in Roswell Park Memorial Institute (RPMI) 1640 media (Gibco), supplemented with 10% (vol/vol) FBS (GEMINI), 1× GlutaMAX™ (Thermo Fisher) at 37°C, 5% (vol/vol) CO_2_, and 90% relative humidity. Plasmid transfection was performed using FuGene^®^ 6 (Promega) for HeLa cells and the Neon transfection system (Life Technologies) for Jurkat cells according to the manufacturers’ protocols. Specifically, HeLa cells were transfected with a total amount of 5 μg plasmids for every 4 × 10^6^ cells and Jurkat cells were transfected with a total amount of 10 μg plasmids for every 2 × 10^6^ cells. For all fluorescence imaging studies, the FP-tagged constructs were co-transfected with their corresponding untagged construct in a 1:4 ratio to rescue the assembly defects seen in cells transfected with FP-tagged constructs only (6). All experiments were performed with cells at passage numbers between 5 and 25.

### Cellular delivery of oligos and self-assembled nanostructures

Following 24 and 48 h transfection of the viral constructs into HeLa cells and Jurkat cells, respectively, oligos and self-assembled nanostructures were nucleofected at the indicated concentrations using the Neon transfection system according to the manufacturer's protocol. In brief, appropriate number of cells were pelleted, resuspended in 1× PBS, and then subjected to nucleofection with nucleofection parameters set at 1005 V with a 35 ms pulse width and 2 pulses total for HeLa cells and 1350 V with a 10 ms pulse width and 3 pulses total for Jurkat cells. Following four washes in culture medium to remove free nucleic acids, the cells were transferred into tissue cultured plates for cell-lysate based analysis or 8-well Lab-Tek Chambered Coverglass (Nunc, Thermo Fisher) previously coated with fibronectin for fluorescence imaging (see below).

### Collection of virus particles and assessment of virus release efficiency by western blot

Virus particles were collected and virus release efficiency was assessed as described previously (6,11), with slight modifications. In brief, WT Gag or Gag mutants expressing HeLa cells (5 × 10^5^ cells) or Jurkat cells (2 × 10^6^ cells) were nucleofected with oligos or nanostructures at the indicated concentrations. At 8 h post-nucleofection for HeLa cells and 48 h post-nucleofection for Jurkat cells, culture supernatants were collected and centrifuged at 1000 × g for 10 min, followed by removal of cell debris and large aggregates with a 0.45 μm syringe filter (Pall Corporation). Thereafter, 2 μl of Dynabeads^®^280 streptavidin (Life Technologies), pre-cleaned with 1× PBS, was added to every mL of the eluent to assist visualization of the pellet after ultracentrifugation at 100 000 × g for 1 h. The pellet (containing both VLPs and the beads) were lysed in lysis buffer (0.5% Triton X-100, 50 mM pH 7.5 Tris–HCl, 300 mM NaCl) supplemented with 10 μl/ml protease inhibitor cocktail (Sigma) for 30 min at 4°C, followed by centrifugation at 21 000 × g for 30 min to remove the beads and membrane debris. To collect cell lysates, the cells were lysed in lysis buffer containing 10 μl/ml protease inhibitor cocktail. Gag from both supernatant and cell lysates was then analyzed by western blot with HIV-Ig (Pooled immunoglobulin from HIV-1-infected patients, obtained from the NIH AIDS Research and Reference Reagent Program). This is followed by determination of Gag levels in the supernatant and in cells via densitometry analysis of Western Blot images using Fiji software (18). VLP release efficiency was calculated as the ratio of supernatant Gag to total (supernatant plus cellular) Gag.

### Immunoprecipitation of Gag-oligo complexes

Immunoprecipitation of Gag-RNA complexes was performed as described previously (9), with modifications. In brief, Gag or Gag mutant expressing HeLa cells (4 × 10^6^ cells) were nucleofected with 5 μM fluorescein-labeled oligos. At 4 h post-nucleofection, cells were fractionated using Minute™ plasma membrane protein isolation (Invent) to separate the PM fraction as per manufacturers’ protocols. Thereafter, the PM fractions were subjected to precleaning with nProtein A-Sepharose™ beads (GE Healthcare) in 500 μl of the IP buffer (100 mM KCl, 5 mM MgCl_2_, 10 mM HEPES, pH 7.05), 0.5% Nonidet P-40, 1 mM DTT, 2 mM vanadyl ribonucleoside complexes solution (Sigma), 10U SUPERase-In™ RNase inhibitor (Promega) and 5 μl protease inhibitor cocktail (Sigma) for 4 h at 4°C. A total of 500 μl of the precleaned lysates was recovered and then supplemented with 8 μg HIV-Ig (Pooled Ig from HIV-1-infected patients obtained from the NIH AIDS Research and Reference Reagent Program). After overnight incubation with gentle mixing at 4°C, 50 μl of 50% (vol/vol) nProtein A-Sepharose™ bead slurry (GE Healthcare) was added to each sample and mixed for 4 h at 4°C. The beads were then washed thrice with lysis buffer with and without 1 M urea. A total of 20% of the sample was spun down at 21 000 × g for 20 min and the beads were resuspended in IP buffer before Western Blot analysis to detect Gag and its mutants with anti-p24 antibodies (EMD Millipore) in each immunoprecipitate. The rest of the sample was spun down at 21 000 × g for 20 min, resuspended and incubated in IP buffer containing 0.1% SDS and 30 μg proteinase K at 50°C for 30 min. Following phenol–chloroform extraction and ethanol precipitation to purify the oligos, dot blot analysis was performed and the oligos were detected using anti-Fluorescein (FAM) antibodies (Abcam).

### Fluorescence *in situ* hybridization

At 8 h post-nucleofection, Gag-EGFP expressing HeLa cells were subjected to fluorescence *in situ* hybridization (FISH) processing as described previously (6,11). In brief, the cells were fixed in 1× PBS solution containing 4% (wt/vol) paraformaldehyde for 20 min at room temperature, washed with 1× PBS for three times, and permeabilized at 4°C in 70% (vol/vol) ethanol overnight. On the next day, the cells were washed thrice with wash buffer containing 2× SSC and 10% (vol/vol) formamide and then incubated in hybridization buffer (10% (wt/vol) dextran sulfate, 2× SSC, 10% (vol/vol) formamide) containing 20 nM singly ATTO647N-labeled oligo probes (/5ATTO647NN/mCmUmCmAmCmGmAmCmAmUmCmAmCmUmUmAmCmGmA, synthesized by Integrated DNA Technologies) against the three loop domains of Tribell and 50 nM singly TAMRA-labeled oligo probes against unspliced HIV-1 viral genomic RNA (gRNA) (11) for 24 h at 37°C in a cell culture incubator. Prior to microscopy imaging, slides were washed thrice with wash buffer and then incubated in wash buffer for 30 min at 37°C, followed by two washes with 2× SSC and a final wash in 1× PBS to remove the unbound probe. Cells were incubated in 1× PBS for imaging.

### Flow cytometric analysis of oligo delivery

Following nucleofection of Gag-EGFP expressing HeLa cells in the presence of 5 μM of Cy5-labeled oligos, the cells were washed four times in culture medium and once in 1× PBS, and analyzed on a FACSVerse flow cytometer (Becton Dickinson) equipped with a 640 nm laser. Flow cytometry data of the Cy5 signal was analyzed using FlowJo (Version 10).

### Fluorescence microscopy

All fluorescence images were acquired on an Olympus IX83 motorized inverted fluorescence microscope equipped with cellTIRF-4Line system and a back-illuminated EMCCD camera (Andor) using CellSens Dimension software. Widefield microscopy imaging was performed using a 100× UPlanSApo 1.4NA objective lens, an MT-20E excitation source (Olympus), and an Olympus MT20 filter set for DAPI, EGFP and TAMRA and a Chroma filter set (ET620/60x, ET700/75m, T660lpxr) for Cy5 or ATTO647N. Three-dimensional image stacks were acquired with 0.3 μm increments in the *z* direction. Image stacks were processed using AutoQuant deconvolution software (MediaCybernetics), followed by maximum-intensity projection using Fiji. spt-PALM imaging was performed using a 100× 1.46 NA total internal reflection objective and 405 nm (100 mW), 488 nm (150 mW) and 561 nm (150 mW) excitation lasers.

### Colocalization analysis

Colocalization between Gag-EGFP and fluorescently-labeled oligos or FISH probes (for FISH imaging of gRNA and Tribell) at PM was determined by first selecting a binary region of interest (ROI) mask in the merged image of the same region acquired in all of the channels using Fiji. Subsequently, the mask was applied to all of the images. The local maxima in each respective ROI was identified using the Find Maxima command available in Fiji. Following determination of the 2D coordinates of the local maxima in the respective channel, the extent of colocalization was determined using a custom MATLAB program. In this context, an EGFP local maximum was treated as a colocalization event if an oligo local maximum was found within a 5 × 5 pixel square centered around the EGFP maximum. The percentage of Gag-EGFP signals that colocalized with oligo signals (%Colocalization EGFP) was calculated by dividing the number of colocalization events by the total number of EGFP local maxima. An oligo local maximum was treated as a colocalization event if an EGFP local maximum was found within a 5 × 5 pixel square centered around the oligo maximum. The percentage of oligo signals that colocalized with Gag-EGFP signals (i.e. %Colocalization TAMRA, %Colocalization Cy5 or %Colocalization ATTO647N) was calculated by dividing the number of colocalization events by the total number of oligo local maxima.

### spt-PALM imaging

Eight-well Lab-Tek Chambered Coverglass (Nunc, Thermo Fisher) were cleaned as previously described (6). Gag-mEOS2 expressing HeLa cells were mocked nucleofected or nucleofected in the presence of 0.33 μM of Tribell and then grown in 8-well chambered coverglass coated with fibronectin. At 16 h post-nucleofection, cells were placed in phenol red-free DMEM containing 25 mM HEPES and 1% FBS and imaged at 37°C. Images were obtained using a 561 nm laser line with intermittent applications of 405 nm activation laser pause to photoconvert mEOS2 probes and recover additional tracks. Time-lapse images were acquired at 20 frames per second.

### spt-PALM analysis

spt-PALM time-lapse images were analyzed to identify trajectories of mEOS2-labeled molecules using the TrackMate plugin of Fiji similar to procedures described previously (6). In brief, localizations of Gag-mEOS2 signals were determined by the Laplacian of Gaussian (LoG) detector (estimated blob diameter = 0.5 μm) and signals within a distance of 500 nm when appearing in consecutive frames or within a distance of 1 μm when having a gap up to 2 frames were assigned to the same trajectory using the simple Linear Assignment Problem (LAP) tracker (linking max distance = 0.5 μm, gap-closing max distance = 1 μm, gap-closing max frame gap = 2). The resulting trajectories containing at least 15 time lags (Δτ) were then selected and analyzed using @msdanalyzer written in MATLAB. Specifically, the Mean Square Displacement (MSD) of all trajectories were calculated and the *D*_eff_ of each trajectory was obtained from a linear fitting of MSD versus Δτ plot, using the first 25% of total time lags, with a fitting threshold of *R*^2^ > 0.8.

### Cluster analysis

Cluster analysis was performed as previously described (6,10), with slight modifications. In brief, Gag-mEOS2 molecules from spt-PALM time-lapse images were first localized using a previously described algorithm written in IDL (Research Systems, Inc.) and the resulting peaks with localization precision 25 nm or less were used for further processing using custom-written MATLAB codes. Specifically, peaks appearing in consecutive frames within a radius of three times the upper limit of the localization precision were considered to arise from the same molecule and were replaced by a single peak with the *x*–*y* coordinate computed as the weighted average of the position coordinates of the contributing peaks. Thereafter, all of the processed peaks were used to construct a composite superresolution image, followed by Hoshen–Kopelman algorithm (19)-based analysis to group connected peaks into the same cluster in the composite image. Next, the size of each cluster was determined by calculating the convex hull (the smallest convex set) for the set of peaks belonging to the cluster. The area of the convex hull and the radius of a circle of equivalent area as the convex hull were used as estimates of cluster area and cluster radius, respectively. The mEOS2 cluster density was calculated by dividing the total number of mEOS2 peaks within the convex hall by the cluster area. The resulting value obtained for each cluster was then normalized to the average density of mEOS2 over the entire PM of the cell. From the set of clusters obtained above, only clusters with a radius less than or equal to 150 nm and density greater than three times the average density of Gag over the PM were considered as Gag assembly platforms arising from oligomerization of Gag at the PM.

### Data analysis

All experiments were repeated at least three times unless otherwise stated. Statistical analyses were performed using either two-tailed Student's *t*-test or one-way ANOVA with post hoc testing of pairwise comparisons using Scheffe's test.

## RESULTS

### Identifying an artificial small RNA ligand of Gag

Nonspecific Gag–RNA interactions are generally accepted as a result of electrostatic binding between the basic residues within Gag's nucleocapsid (NC) domain and the negatively charged RNA phosphate backbone. Nonetheless, it is still unclear whether Gag, when engaged in electrostatic RNA binding, can display a binding preference for structured over unstructured elements as observed with other RNA binding proteins (20,21). Therefore, as a starting point, we sought to investigate whether a simple secondary structure, i.e. a small stem–loop (STL) motif, can be a prominent ligand of Gag in cells, inspired by studies indicating that microRNAs can form a stem–loop structure (22,23) and results from chemical probing-based RNA structure analysis of gRNA extracted from HIV-1 virus particles revealing the presence of many secondary structures throughout the gRNA (24).

To this end, we synthesized a simple STL oligo, termed STL1 (Supplementary Table S1), based on the following criteria: (i) nucleotides modified by 2′-*O*-methylation, which is a naturally occurring modification in biological systems that provides resistance to intracellular nuclease digestion (25); (ii) a non-endogenous sequence, neither present in the viral nor human genome, to avoid sequence-specific Gag binding or antisense responses; (iii) size similar to microRNA, established based on previous findings that microRNAs are prominent ligands of Gag in cells (9,11); (iv) structure mimicking the stem–loop structure of a previously reported oligo (i.e. molecular beacon, MB) that can be maintained in cells (26,27) (see http://www.molecular-beacons.org/MB_publications.html#cap1 for a comprehensive guideline on the design of MB). To assess Gag-STL1 interaction, HeLa cells were first transfected with HIV-1 pNL43 derivative constructs (Supplementary Figure S1), followed by delivery of STL1 via nucleofection (referred to as STL1+ cells). For comparison, cells that were nucleofected with an unstructured analogue (UN1) of STL1, denoted as UN1+ cells, or nucleofected in the absence of oligos, denoted as mock-nucleofected cells, were also prepared. It was hypothesized that comparison of the Gag binding capacity of STL1 and UN1 in cells should help determine whether our simple stem–loop design could be a prominent ligand of Gag and a potent inhibitor of HIV-1 assembly.

**Figure 1.**
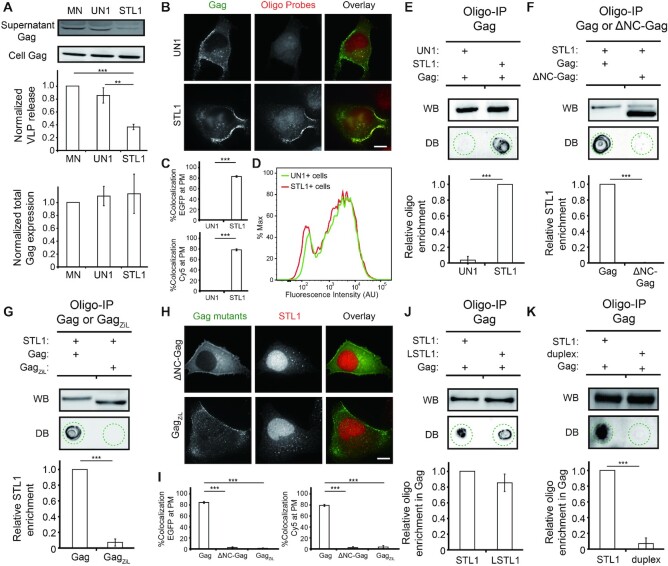
Gag preferentially interacts with STL1 over UN1 at PM via its nucleocapsid (NC) domain and that the interaction inhibits HIV-1 viral production. (**A**) Assessment of virus release efficiency and total Gag expression levels by western blot in mock-nucleofected (MN), UN1 + or STL1 + cells expressing Gag (through pNL43ΔPolΔEnv-Gag transfection). Results were normalized to the virus release and total Gag expression levels of MN cells. (**B**) Representative images of Gag-EGFP (through co-transfection of pNL43ΔPolΔEnv-Gag-EGFP and pNL43ΔPolΔEnv-Gag at a 1:4 ratio) and UN1 or STL1 (tagged with a Cy5 fluorophore at the 5′-end) in UN1 + or STL1 + cells expressing Gag-EGFP. (**C**) The percentage of EGFP signals that were colocalized with Cy5 signals (%Colocalization EGFP) and the percentage of Cy5 signals that were colocalized with EGFP signals (%Colocalization Cy5) were calculated for cells from (B) on a cell-by-cell basis. **(D)** Flow cytometric measurements of Cy5 fluorescence in UN1 + or STL1 + cells from (B). The flow cytometry histograms correspond to the Cy5 signal of 10 013 single UN1 + cells (green) and 10 144 single STL1 + cells (red). (**E–G**) Immunoprecipitation of the oligos in complex with Gag (through pNL43ΔPolΔEnv-Gag transfection) or ΔNC-Gag (pNL43ΔPolΔEnv-ΔNC-Gag transfection), or Gag_ZiL_ (through pNL43ΔPolΔEnv-Gag_ZiL_ transfection) in the PM fraction of UN1 + and STL1 + cells (the oligos were tagged with a FAM fluorophore at the 5′-end). Immunoprecipitated Gag or the NC mutant was detected by western blot (WB) and oligos in each immunoprecipitate was detected by dot blot (DB) (see Materials and Methods). Dashed circles indicate the location of the dots. (**H**) Representative images of STL1 (tagged with a Cy5 fluorophore at the 5′-end) in STL1 + cells expressing ΔNC-Gag-EGFP (through co-transfection of pNL43ΔPolΔEnv-ΔNC-Gag-EGFP and pNL43ΔPolΔEnv-ΔNC-Gag at a 1:4 ratio) or Gag_ZiL_-EGFP (through co-transfection of pNL43ΔPolΔEnv-Gag_ZiL_-EGFP and pNL43ΔPolΔEnv-Gag_ZiL_ at a 1:4 ratio). (**I**) Cell-by-cell analysis of %Colocalization EGFP and %Colocalization Cy5 at the PM of the cells from (H), plotted together with the data acquired for Gag and STL1 at the PM of STL1 + cells as in (C). (**J**, **K**) Oligo immunoprecipitation experiments in STL1 + cells, LSTL + cells, and STL1 duplex + cells expressing Gag (through pNL43ΔPolΔEnv-Gag transfection). For (A), (E-G) and (J, K), data represent mean ± SD of three replicate experiments. For (C) and (I), data represent mean ± SEM of at least 15 cells. Scale bar = 10 μm. Asterisks indicate *P*-values (** *P* < 0.01, *** *P* < 0.001).

Virus release assays showed that, compared with mock-nucleofected cells, STL1+ cells but not UN1+ cells exhibited reduced capacity to form HIV-1 particles (Figure 1A). Total Gag expression was similar in the three samples, suggesting that the observed reduction in viral production in STL1+ cells was not caused by silencing of viral genes. Rather, the observed viral blockade appeared to arise as a result of Gag–STL1 complexes interfering with Gag multimerization at the PM since, when viewed by fluorescence microscopy, STL1 but not UN1 (Cy5-labeled) could colocalize extensively with clusters formed by Gag tagged with EGFP (co-transfected with the untagged construct in a 1:4 ratio to rescue the assembly defects seen in cells transfected with FP-tagged constructs only (6)) (Figure 1B and C), while there is no detectible difference in the amount of the two oligos in cells as indicated by flow cytometry (Figure 1D). Evidence that STL1 binds more readily to Gag than UN1 came from oligonucleotide immunoprecipitation (oligo-IP) experiments showing that the quantity of oligos retrieved by immunoprecipitating Gag from STL1+ cells was much greater than that observed during immunoprecipitation of Gag from UN1+ cells (Figure 1E). The observed Gag-STL1 binding occurs through Gag NC, as previously established Gag mutants harboring a deletion of NC (i.e. ΔNC-Gag) or an isoleucine zipper motif in place of NC (i.e. Gag_ZiL_) (28) cannot interact with STL1 at PM when investigated by oligo-IP (Figure 1F and G) and fluorescence microscopy imaging (Figure 1H and I). Moreover, compared with STL1, an STL1-based oligo with a longer stem (denoted as LSTL1) exhibited similar Gag binding (Figure 1J), whereas the prehybridized duplex formed from STL1 and its complementary RNA target exhibited no detectable Gag binding (Figure 1K), consistent with previous studies showing Gag binds poorly to duplex nucleic acids (29). Thus, we conclude that STL1 is an efficient ligand design of Gag that can interfere with virus assembly through binding with NC. Finally, oligo-IP experiments and virus release assays performed with three other sets of non-endogenous STLs with similar structures as STL1 and their corresponding UN analogues (Supplementary Table S1 and Supplementary Figures S2–S4) also showed that STL oligos exhibited enhanced Gag binding as well as enhanced capacity to block HIV-1 particle formation compared with the UN analogues. Since the four STL oligos identified as prominent Gag binders have no sequence homology, we conclude that Gag has the ability to bind small STL oligos irrespective of oligonucleotide sequence and the resulting Gag-STL complexes can inhibit HIV-1 viral production.

### Engineering higher-order RNA nanostructures to block HIV-1 assembly

**Figure 2.**
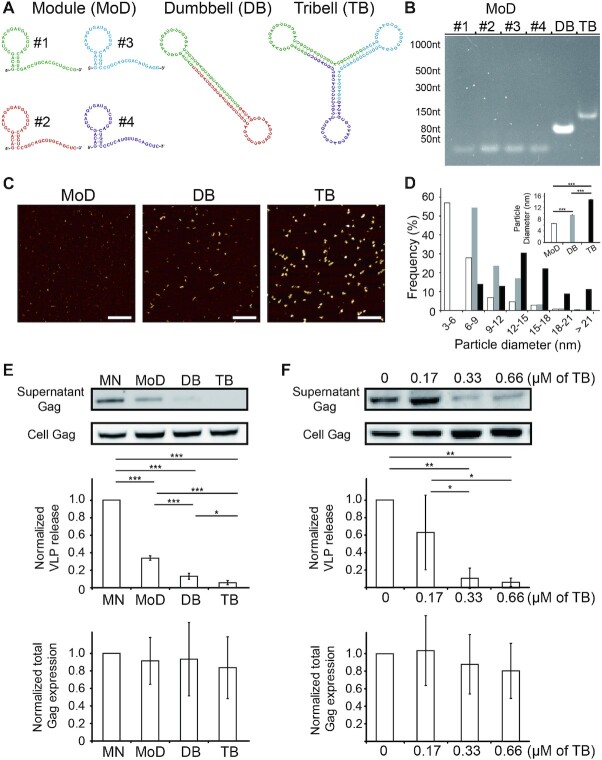
Assembling RNA nanostructures from stem–loop forming (STL) oligos to inhibit HIV-1 viral production. (**A**) Design and assembly of Dumbbell (DB) and Tribell (TB) structures from STL module oligos (MoDs). DB is assembled from MoD #1 and MoD #2 and TB is assembled from MoD #1, MoD #3 and MoD #4. (**B**) Representative 6% native PAGE gel confirming step-wise assembly of the higher-order structures from MoDs. (**C**, **D**) Representative AFM topography images and size analysis of the MoD (i.e. MoD #1), DB and TB designs. All images were adjusted to the same maximum and minimum height scales. Scale bar = 100 nm. The histogram shows the distribution of particle diameters of MoD #1 (*n* = 1134), DB (*n* = 1271) and TB (*n* = 1202). *Inset* shows mean ± SEM particle diameter. (**E**) Assessment of virus release efficiency and total Gag expression levels by western blot in mock-nucleofected (MN), MoD+, DB+ or TB+ cells expressing Gag (through pNL43ΔPolΔEnv-Gag transfection). Results were normalized to the virus release and total Gag expression levels of MN cells. (**F**) Assessment of virus release efficiency and total Gag expression levels by western blot in cells expressing Gag (through pNL43ΔPolΔEnv-Gag transfection) followed by nucleofection of different quantities of TB. Results were normalized to the virus release and total Gag expression levels of MN cells. For (E) and (F), data represent mean ± SD of three and four replicate experiments, respectively. Asterisks indicate *P*-values (*P < 0.05, ** *P* < 0.01, *** *P* < 0.001).

With unique properties such as specific base pairing and predictable structure and size, RNA molecules have been used as raw materials to fabricate complex functional nanostructures via programmed self-assembly methods in RNA nanotechnology (12–15). This led us to investigate whether nonspecific STL motifs, capable of inhibiting viral production as demonstrated above, may be used as modules to construct higher-order nanostructures that can more potently inhibit viral production. To test this idea, we synthesized a set of non-endogenous STL forming oligos with one arm of the stem being significantly longer than the other, and used them as modules (denoted as the Module design) to form a double and a triple STL architectures, denoted as Dumbbell and Tribell, respectively (Figure 2A; see Materials and Methods). Native PAGE analysis and 2D topographical measurements by atomic force microscopy (AFM) both revealed detectible size differences among the three designs (Figure 2B–D), confirming successful step-wise self-assembly.

Compared with mock-nucleofected cells, HeLa cells nucleofected with the three designs at concentrations corresponding to the same number of STLs (i.e. 1 μM for Module, 0.5 μM for Dumbbell and 0.33 μM for Tribell) all exhibited reduced viral production, while total Gag expression was similar in the four samples (Figure 2E). Thus, similar to the parental STL design, the new designs can inhibit HIV-1 particle production by a mechanism not involving gene silencing. Notably, there was a difference in the viral blockade capacity among the three designs, with cells nucleofected with Module, Dumbbell and Tribell exhibiting 34.0% ± 2.5%, 13.3% ± 3.5% and 6.0% ± 2.4% virus release efficiency, respectively (Figure 2E). Moreover, analogous experiments performed in Jurkat CD4 + T lymphocytes also showed that the Tribell structure could result in the highest viral blockade among the three designs (Supplementary Figure S5). Importantly, the Tribell structure, despite being larger in size and nucleofected at lower concentrations, can more potently inhibit viral production than the Module and Dumbbell structures in both HeLa and Jurkat cells (Figure 2E and Supplementary Figure S5). This indicates that multiple STLs, when combined to form a higher-order nanostructure, can more potently inhibit viral production than when the STLs are used as separate entities. Finally, Tribell could inhibit viral production in a dose-dependent manner (Figure 2F and Supplementary Figure S6). Altogether, these results establish programmed self-assembly as a vital approach for tunable and modular construction of RNA nanostructures that can potently inhibit HIV-1 viral budding and release.

### Deciphering the mechanism of nanostructure-mediated viral blockade

To gain mechanistic insights underpinning the observed viral blockade by the RNA nanostructures, we measured the mobility of Gag molecules tagged with mEOS2 (Supplementary Figure S1; see Materials and Methods) at PM of mock-nucleofected cells and HeLa cells that were nucleofected with Tribell (referred to as Tribell + cells) by single particle tracking photoactivated localization microscopy (spt-PALM), which can reveal dynamics of individual proteins within subdiffraction space (30). It was hypothesized that under conditions where Gag can efficiently multimerize, Gag should wind up into tight clusters that are immobile. By contrast, if Gag multimerization is disrupted by Tribell, Gag should move more freely at PM because they cannot efficiently interact with one another to form clusters.

**Figure 3.**
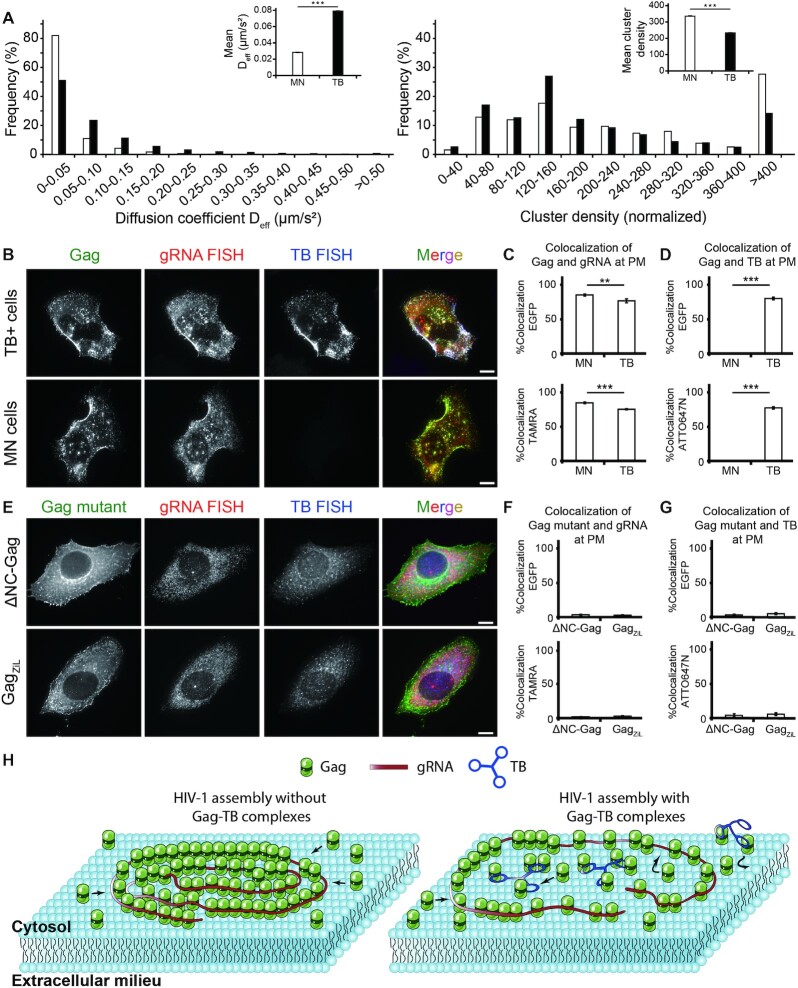
Tribell (TB) interacts with the nucleocapsid (NC) domain of Gag to disrupt viral genomic RNA (gRNA)-mediated Gag multimerization at the PM. (**A**) spt-PALM measurements and cluster analysis of single Gag-mEOS2 molecules within Gag complexes diffusing across the PM in mock-nucleofected (MN) and Tribell (TB)+ cells transfected with pNL43ΔPolΔEnv-Gag-mEOS2 and pNL43ΔPolΔEnv-Gag at a 1:4 ratio. The left histogram shows the distribution of diffusion coefficients (*D*_eff_) of single Gag molecules in MN cells (*n* = 13 780 tracks) and in TB+ cells (*n* = 17 950 tracks). *Inset* shows the mean ± SEM diffusion coefficients. The right histogram shows the cluster density distribution of Gag at the PM of MN and TB+ cells (*n* = 18 449 clusters for MN cells, *n* = 7978 clusters for TB+ cells). For each cell, the cluster densities were normalized with respect to the mean density of Gag at the PM. *Inset* shows the mean ± SEM cluster densities. Data were acquired from 18 MN cells and 15 TB+ cells. (**B**) Representative images of Gag-EGFP, gRNA (detected by TAMRA-labeled FISH probes) and TB (detected by ATTO647N-labeled FISH probes) in MN or TB+ cells transfected with pNL43ΔPolΔEnv-Gag-EGFP and pNL43ΔPolΔEnv-Gag at a 1:4 ratio. **(C, D)** Colocalization analysis between Gag and gRNA or TB at PM of cells from (B). (**C**) Colocalization between Gag and gRNA. The percentage of EGFP signals that were colocalized with gRNA FISH signals (%Colocalization EGFP) and the percentage of gRNA FISH signals that were colocalized with EGFP signals (%Colocalization TAMRA) were calculated. (**D**) Colocalization between Gag-EGFP and TB. The percentage of EGFP signals that were colocalized with TB FISH signals (%Colocalization EGFP) and the percentage of TB FISH signals that were colocalized with EGFP signals (%Colocalization ATTO647N) were calculated. Data represent mean ± SEM of 38 MN cells and 25 TB+ cells. (**E**) Representative images of each Gag mutant ΔNC-Gag-EGFP or Gag_ZiL_-EGFP, gRNA (detected by TAMRA-labeled FISH probes) and TB (detected by ATTO647N-labeled FISH probes) in TB + cells transfected with either pNL43ΔPolΔEnv-ΔNC-Gag-EGFP or pNL43ΔPolΔEnv-Gag_ZiL_-EGFP in a 1:4 ratio with the respective untagged construct. (**F, G**) Colocalization analysis between each Gag mutant and gRNA or TB at PM of cells from (E). (**F**) Colocalization analysis between each Gag mutant and gRNA or TB. %Colocalization EGFP and %Colocalization TAMRA were calculated. (**G**) Colocalization analysis between each Gag mutant and TB. %Colocalization EGFP and %Colocalization ATTO647N were calculated. Data represent mean ± SEM of 20 ΔNC-Gag-EGFP expressing cells and 17 Gag_ZiL_-EGFP expressing cells. (**H**) Schematic model of Gag–TB complexes interfering with gRNA-mediated Gag multimerization at PM. In the absence of Gag–TB complexes, Gag and the gRNA form stable complexes at the PM, resulting in viral budding. In the presence of Gag–TB complexes, the complexes interfere with gRNA-mediated Gag multimerization, resulting in inhibition of HIV-1 assembly and particle production. Scale bar = 10 μm. Asterisks indicate *P*-values (** *P* < 0.01, *** *P* < 0.001).

Supporting this possibility, Gag's statistical distribution of diffusion coefficient (*D*_eff_) shifted to more rapid diffusion in Tribell + cells compared with mock-nucleofected cells. The mean *D*_eff_ of Gag at PM was 0.0283 ± 0.0004 μm^2^/s in mock-nucleofected cells and 0.0789 ± 0.0006 μm^2^/s in Tribell + cells (Figure 3A). Additionally, PALM cluster analysis revealed increased tendency of Gag to form clusters in mock-nucleofected cells than in Tribell+ cells (Figure 3A). Specifically, the mean Gag cluster size in mock-nucleofected cells was 1.32 times greater than that in Tribell+ cells, with 63.0% of Gag molecules associated in clusters in mock-nucleofected cells compared with 14.6% in Tribell+ cells. Within each cluster, the average density of Gag was 1.44 times greater in mock-nucleofected than in Tribell+ cells. Hence, Gag molecules exhibit greater mobility and less tendency to form clusters at the PM of Tribell+ cells. The impaired Gag clustering in Tribell+ cells was like a result of Tribell competing with gRNA for electrostatic Gag binding, as Gag could colocalize extensively with Tribell while colocalizing with gRNA (Figure 3B–D). Furthermore, the observed Gag-Tribell colocalization was accompanied by a slight but notable reduction in Gag-gRNA colocalization (assessed by fluorescence *in situ* hybridization, FISH) (Figure 3B–D). This suggests that, besides blocking Gag from binding with the gRNA non-Ψ region that is considered largely electrostatic, Tribell could to some extent prevent Gag from binding electrostatically with Ψ, since electrostatic binding could also contribute to Gag-Ψ interactions at physiologically relevant salt concentrations (3). Interactions with both Tribell and gRNA were mainly mediated by the Gag NC domain, as Gag-EGFP lacking the NC domain could not colocalize with both Tribell and gRNA at PM (Figure 3E–G). Thus, when Gag interacts with the STL-decorated nanostructure at PM, its interaction with gRNA is inhibited and its mobility increases at PM, Gag coalescence and multimerization into tight clusters is impeded, and viral budding is inhibited (Figure 3H).

## DISCUSSION

Currently, the majority of RNA-guided therapeutics have been developed by harnessing antisense or aptamer-based recognition (31,32). Little attention has been given to developing strategies that can intervene electrostatic interactions that are increasingly discovered to be key determinants of various pathological conditions (33–37). This work establishes a programmed self-assembly method for developing such agents in a controlled and systematic fashion that can potently block the assembly of HIV-1 virus particles mediated by electrostatic RNA-protein interactions in cells. In particular, through analysis of four different STL and UN sequences (Supplementary Table S1), we first identified that short STL-forming oligos modified by 2′-*O*-methylation, regardless of sequence, can disrupt viral particle formation at PM by binding with Gag (Figure 1A and E and Supplementary Figures S2–S4). This is followed by experiments showing self-assembly of STL oligos into higher-order nanostructures that can more potently inhibit viral assembly than single STLs. It is worth noting that this proof-of-concept study presents a previously undescribed property of Gag that during electrostatic binding with RNA the protein exhibits a binding preference for simple STL motifs, and is the first report of anti-HIV-1 assembly strategy based on artificial small RNAs as well as RNA nanostructures. Thus, our approach is different from antisense-based approaches that use a specific sequence to target a specific RNA, or aptamer-based approaches that use systematic evolution of ligands by exponential enrichment (SELEX) to derive a specific sequence-structure motif that can bind a target protein (38–42). Experiments aiming to define a more efficient and effective STL design through varying the STL stem and loop sizes, as well as comparing the resulting design with some of the previously reported aptamers raised against Gag or its components (43–49), are currently underway.

We suggest that the self-assembled RNA nanostructures, despite being larger and introduced into cells at a lower amount, could more potently inhibit virus production than the module designs because the higher-ordered nanostructures are too large to traverse through nuclear pore channels that are only ∼5 nm in diameter (50), and are thus less prone to nuclear sequestration often observed with small oligonucleotides (51) (compare Figures 1B and 3B). As such, the RNA nanostructures may be more readily available to interact with Gag to prevent its multimerization at PM than the single STL of the modules. Additionally, it is possible that the larger structures, while enabling more Gag molecules to bind, can also help stabilize Gag binding due to Gag's ability to bind RNA in a cooperative manner (3). Future studies will be required to test if retaining single STLs in the cytoplasm (e.g., through conjugation with a large nanocrystal (52) or simply increasing the size of single STLs with unstructured RNA), incorporating more STLs, or enhancing the biostability (e.g., via incorporation of chemically modified nucleotides that are more nuclease-resistant than the 2′-*O*-methyl RNA used in this study) can lead to a more potent nanostructure.

We should also mention that compared with the widely used antisense and aptamer-based methods that in the case of HIV-1 might only be potent against a particular viral strain, the self-assembled RNA nanostructures described here have the potential to block the electrostatic Gag–RNA interaction feature that is highly conserved across different strains. Therefore, we envision our system could serve as a generalized supportive platform for combined use with various strain-specific antisense or aptamer molecules to achieve an additive anti-HIV-1 effect. Finally, given the ever-growing discovery that RNA-mediated protein assembly processes may contribute to the pathogenesis of diseases such as prion diseases, Alzheimer's disease and COVID-19 (33–37), our results suggest the possibility of using RNA nanostructures to disrupt these processes as a potent therapeutic approach.

## DATA AVAILABILITY

The raw flow cytometry data has been deposited on FlowRepository (repository ID: FR-FCM-Z4KW).

## Supplementary Material

gkab1282_Supplemental_File
