# Supplementary material for: Rational design of self-assembled RNA nanostructures for HIV-1 virus assembly blockade
Source: Nucleic Acids Res. 2021 Dec 30;50(8):e44. doi: 10.1093/nar/gkab1282 (PMC9071489; doi:10.1093/nar/gkab1282)
Supplement: gkab1282_Supplemental_File [file gkab1282_supplemental_file.pdf]

SUPPLEMENTARY MATERIALS

Rational design of self-assembled RNA nanostructures for HIV-1 virus assembly blockade

Na Qu[1], Yachen Ying[1,2], Jinshan Qin[1], and Antony K. Chen[1,*]

[1]Department of Biomedical Engineering, College of Future Technology, Peking University, Beijing 100871, China

[2]Department of Biomedical Engineering, College of Engineering, Peking University, Beijing 100871, China

*ADDRESS FOR CORRESPONDENCE:

ANTONY K. CHEN

Department of Biomedical Engineering

College of Future Technology, Peking University

No. 5 Yiheyuan Road.

Haidian District, Beijing 100871, China

Tel/Fax: +86 10 6276 8343

EMAIL: chenak@pku.edu.cn

Table S1.

Oligo name	Sequence
STL1	<u>mGmUmC mAmC</u>mC mUmCmA mGmCmG mUmAmA mGmUmG mAmUmG mUmC<u>mG mUmGmA mC</u>
STL2	<u>mCmUmU mCmG</u>mU mCmCmA mCmAmA mAmCmA mCmAmA mCmUmC mC<u>mUmG mAmAmG</u>
STL3	<u>mCmUmA mGmC</u>mU mCmUmA mAmAmU mCmAmC mUmAmU mCmCmU mCmGmC <u>mGmCmU mAmG</u>
STL4	<u>mGmGmU mCmG</u>mU mUmGmU mAmGmG mUmUmC mCmAmC mUmGmG mUmU<u>mC mGmAmC mC</u>
STL1 with longer stem length	<u>mGmUmC mAmCmG mUmCmA mC</u>mCmU mCmAmG mCmGmU mAmAmG mUmGmA mUmGmU mC<u>mGmU mGmAmC</u> <u>mGmUmG mAmC</u>
UN1	mCmUmC mAmGmC mGmUmA mAmGmU mGmAmU mGmUmC mGmU
UN2	mCmUmU mCmGmU mCmCmA mCmAmA mAmCmA mCmAmA mCmUmC mCmUmG
UN3	mCmUmA mCmAmU mCmUmA mAmAmU mCmAmC mUmAmU mCmCmU mCmGmC mGmCmU mAmA
UN4	mCmUmA mCmAmU mUmGmU mAmGmG mUmUmC mCmAmC mUmGmG mUmUmGmCmU mAmA
STL1 target RNA (for duplex)	rUrGrG rArCrA rUrCrA rCrUrU rArCrG rCrUrG rArGrU rA
Module 1 (MoD #1)	<u>mGmAmC mAmG</u>mC mGmUmA mAmGmU mGmAmU mGmUmC mGmUmG mA<u>mCmU mGmUmC</u> mGmAmG mCmUmG mCmAmC mGmCmU mGmCmC mG
Module 2 (MoD #2)	<u>mGmAmC mAmG</u>mC mGmUmA mAmGmU mGmAmU mGmUmC mGmUmG mA<u>mCmU mGmUmC</u> mCmGmG mCmAmG mCmGmU mGmCmA mGmCmU mC
Module 3 (MoD #3)	<u>mGmAmC mAmG</u>mC mGmUmA mAmGmU mGmAmU mGmUmC mGmUmG mA<u>mCmU mGmUmC</u> mCmGmG mCmAmG mCmGmA mCmAmU mGmAmG mG
Module 4 (MoD #4)	<u>mGmAmC mAmG</u>mC mGmUmA mAmGmU mGmAmU mGmUmC mGmUmG mA<u>mCmU mGmUmC</u> mCmCmU mCmAmU mGmUmU mGmCmA mGmCmU mC

Table S1. Nonsense oligonucleotides (oligos) used in this study. Underlined letters indicate the stem. m represents 2’-O-methyl RNA modification. r represents no modification.

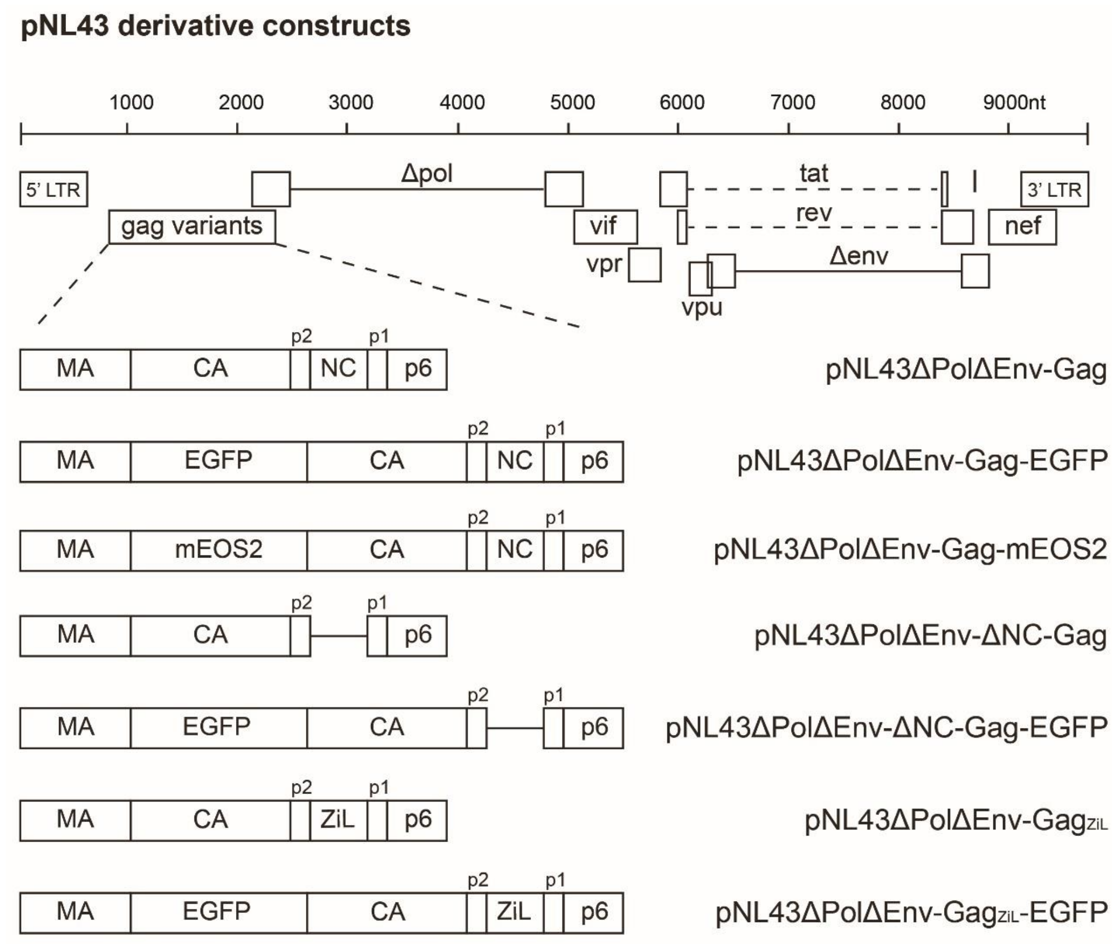

Figure S1. Schematic representation of the plasmid constructs used in this study.

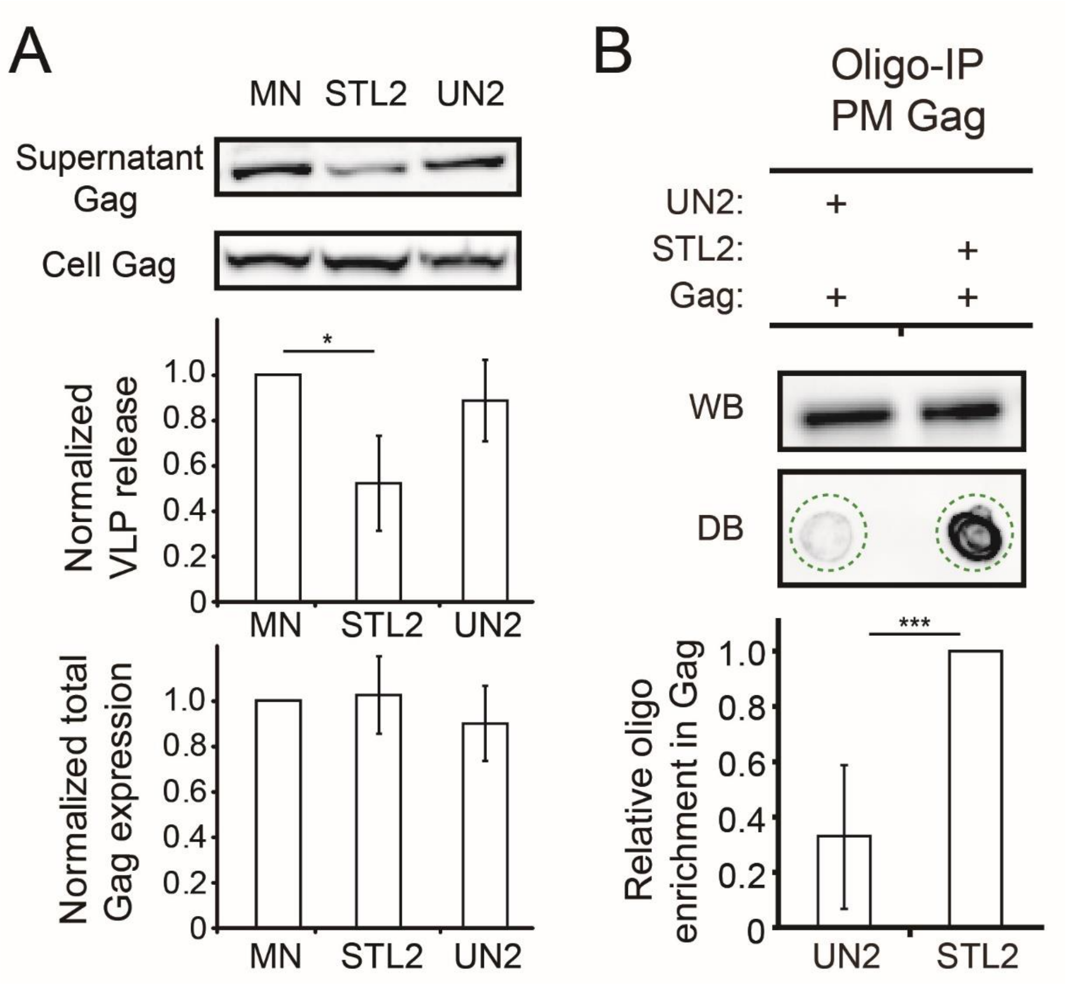

Figure S2. Gag preferentially interacts with STL2 over UN2 at PM and the interaction inhibits HIV-1 viral production. (A) Assessment of virus release efficiency and total Gag expression levels by western blot in mock-nucleofected (MN), UN2+ or STL2+ cells expressing Gag (through pNL43ΔPolΔEnv-Gag transfection). Results were normalized to the virus release and total Gag expression levels of MN cells. **(B)** Immunoprecipitation of the oligos in complex with Gag (through pNL43ΔPolΔEnv-Gag transfection) in the PM fraction of UN2+ and STL2+ cells (the oligos were tagged with a FAM fluorophore at the 5'-end). Immunoprecipitated Gag was detected by western blot (WB) and oligos in each immunoprecipitate was detected by dot blot (DB) as described in the Materials and Methods. Dashed circles indicate the location of the dots. Data represent mean ± SD of three replicate experiments. Asterisks indicate *P*-values (*$P < 0.05$, *** $P < 0.001$).

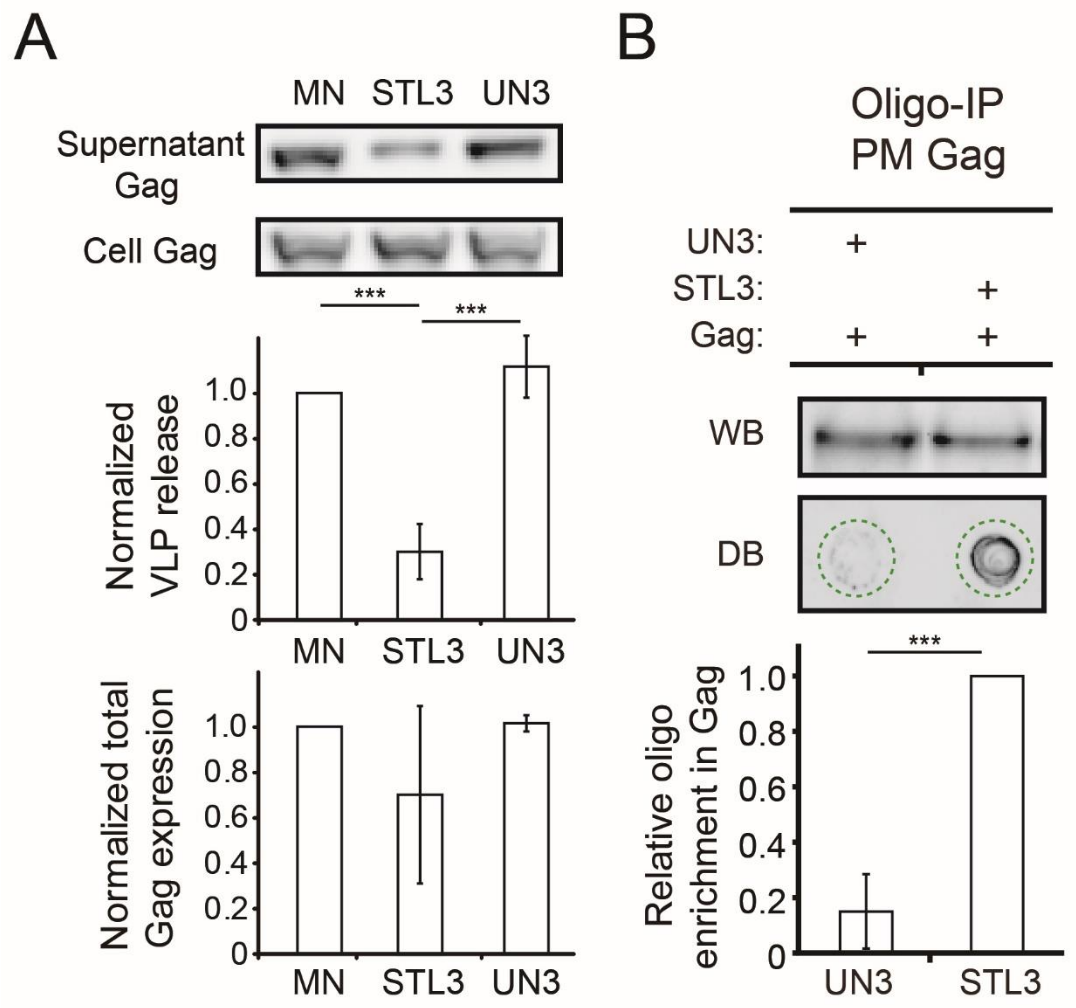

Figure S3. Gag preferentially interacts with STL3 over UN3 at PM and the interaction inhibits HIV-1 viral production. (A) Assessment of virus release efficiency and total Gag expression levels by western blot in mock-nucleofected (MN), UN3+ or STL3+ cells expressing Gag (through pNL43ΔPolΔEnv-Gag transfection). Results were normalized to the virus release and total Gag expression levels of MN cells. **(B)** Immunoprecipitation of the oligos in complex with Gag (through pNL43ΔPolΔEnv-Gag transfection) in the PM fraction of UN3+ and STL3+ cells (the oligos were tagged with a FAM fluorophore at the 5'-end). Immunoprecipitated Gag was detected by western blot (WB) and oligos in each immunoprecipitate was detected by dot blot (DB) as described in the Materials and Methods. Dashed circles indicate the location of the dots. Data represent mean ± SD of three replicate experiments. Asterisks indicate $P < 0.001$.

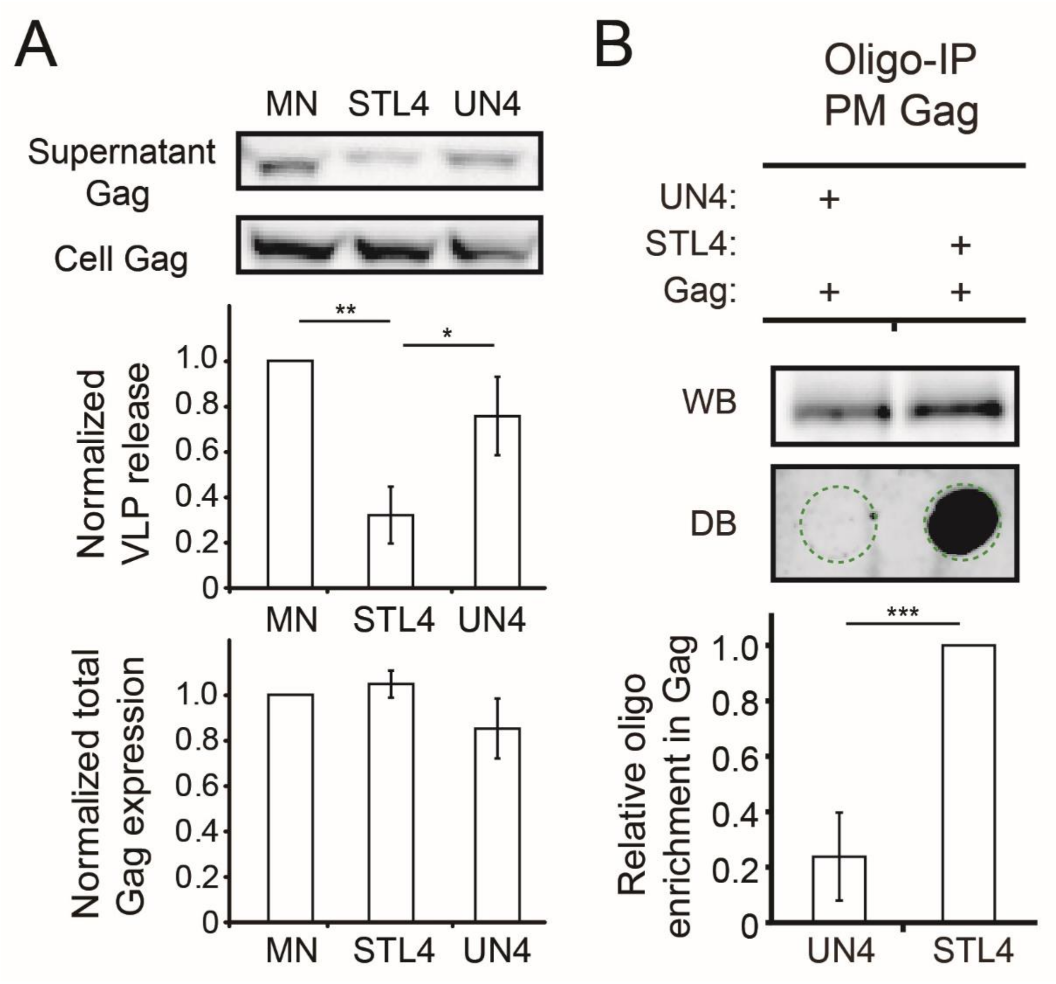

Figure S4. Gag preferentially interacts with STL4 over its unstructured analog (UN4) at PM and the interaction inhibits HIV-1 viral production. (A) Assessment of virus release efficiency and total Gag expression levels by western blot in mock-nucleofected (MN), UN4+ or STL4+ cells expressing Gag (through pNL43ΔPolΔEnv-Gag transfection). Results were normalized to the virus release and total Gag expression levels of MN cells. **(B)** Immunoprecipitation of the oligos in complex with Gag (through pNL43ΔPolΔEnv-Gag transfection) in the PM fraction of UN1+ and STL1+ cells (the oligos were tagged with a FAM fluorophore at the 5'-end). Immunoprecipitated Gag was detected by western blot (WB) and oligos in each immunoprecipitate was detected by dot blot (DB) as described in the Materials and Methods. Dashed circles indicate the location of the dots. Data represent mean ± SD of three replicate experiments. Asterisks indicate *P*-values (*$P < 0.05$, ** $P < 0.01$, *** $P < 0.001$).

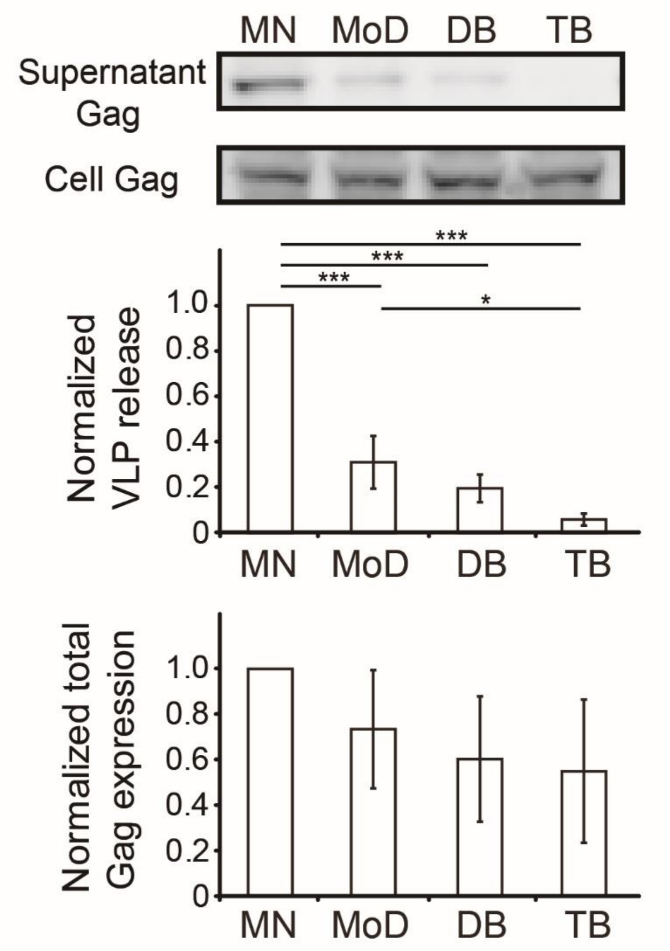

Figure S5. The impact of stem-loop (STL) forming oligonucleotides and self-assembled STL-decorated nanostructures on HIV-1 viral production in Jurkat cells. Virus release efficiency and total Gag expression levels were assessed in Jurkat cells expressing Gag (through pNL43ΔPolΔEnv-Gag transfection) that are nucleofected in the absence of oligos (mock-nucleofected, MN) or in the presence of 1 µM of STL module oligo #1 (MoD #1), 0.5 µM of dumbbell (DB) oligos and 0.33 µM of Tribell (TB) oligos. Results were normalized to the virus release and total Gag expression levels of MN cells. Data represent mean ± SD of three replicate experiments. Asterisks indicate *P*-values (*$P < 0.05$, *** $P < 0.001$).

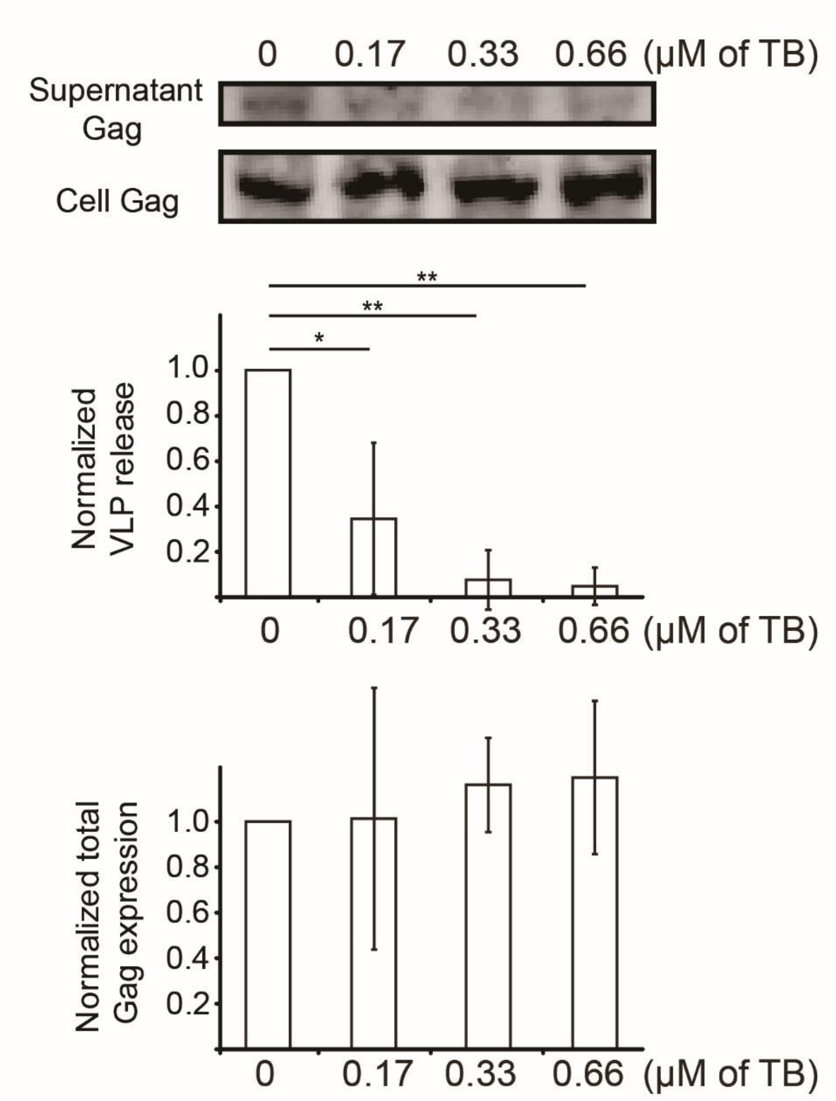

Figure S6. Tribell (TB) nanostructures inhibit HIV-1 viral production in a dose-dependent manner in Jurkat cells. Virus release efficiency and total Gag expression levels were assessed in Jurkat cells transfected with pNL43ΔPolΔEnv-Gag followed by nucleofection in the presence of different quantities of TB. Results were normalized to the virus release and total Gag expression levels of cells microporated with no oligos (i.e., mock-nucleofected cells). Data represent mean ± SD of four replicate experiments. Asterisks indicate *P*-values (*$P < 0.05$, ** $P < 0.01$).
